# Association of the uric acid to albumin ratio with early glycemic disturbances beyond conventional OGTT criteria

**DOI:** 10.1186/s12902-026-02314-x

**Published:** 2026-05-12

**Authors:** Irem Sonmezoglu Kutuk, Sabri Engin Altintop, Ethem Turgay Cerit, Mehmet Muhittin Yalcin, Alev Eroglu Altinova, Mujde Akturk, Fusun Toruner, Mehmet Ayhan Karakoc

**Affiliations:** Department of Internal Medicine, Division of Endocrinology and Metabolism, Gazi University Faculty of Medicine, Ankara, Turkey

**Keywords:** Oral glucose tolerance test, Uric acid to albumin ratio, Intermediate hyperglycemia, Prediabetes, Glycemic dynamics

## Abstract

**Background:**

Conventional interpretation of the oral glucose tolerance test (OGTT) relies on fasting and 2-hour plasma glucose values and may not fully reflect early disturbances in glucose metabolism. Intermediate OGTT time points, particularly 1-hour and peak glucose levels, have been associated with early alterations in insulin sensitivity and β-cell function. The uric acid-to-albumin ratio (UAR) has been proposed as a composite metabolic marker; however, its relationship with OGTT-derived glycemic dynamics remains unclear.

**Methods:**

In this retrospective observational study, 1,097 adults undergoing a standard 75-g OGTT were included. Glycemic status was classified according to ADA criteria. Intermediate hyperglycemia was primarily defined as a 60-minute plasma glucose ≥ 155 mg/dL, based on previously reported risk-associated thresholds. UAR was calculated from serum uric acid and albumin levels. Associations between UAR and glycemic parameters were evaluated using correlation analyses and multivariable logistic regression models adjusted for age, sex, BMI, renal function, and lipid parameters. Multivariable analyses were performed in a complete-case subset of participants with available covariate data (*n* = 378).

**Results:**

Individuals with intermediate hyperglycemia (60-minute glucose ≥ 155 mg/dL) had higher UAR levels compared with those without such abnormalities (*p* < 0.001). UAR showed significant correlations with OGTT-derived parameters, including glucose excursion, time to peak glucose, and OGTT area under the curve. In multivariable analyses, UAR was not independently associated with prediabetes (*p* = 0.228), but remained independently associated with 60-minute glucose ≥ 155 mg/dL (OR 1.13 per 0.1-unit increase, 95% CI 1.03–1.24; *p* = 0.006). Discriminative performance for prediabetes was modest (AUC = 0.62).

**Conclusions:**

UAR is associated with intermediate glycemic abnormalities during OGTT in this cohort. While not a standalone diagnostic marker, it may provide complementary metabolic information in relation to early OGTT-derived disturbances. Further prospective studies are required to clarify its potential role in early dysglycemia.

**Supplementary Information:**

The online version contains supplementary material available at 10.1186/s12902-026-02314-x.

## Introductıon

Diabetes mellitus represents a major global public health challenge, with a steadily increasing prevalence worldwide, as highlighted by recent estimates from the International Diabetes Federation [1]. This growing burden underscores the importance of early identification of dysglycemia and timely intervention to prevent progression to overt diabetes. The oral glucose tolerance test (OGTT) is widely used for the assessment of glucose metabolism and the diagnosis of prediabetes and diabetes mellitus; however, conventional clinical interpretation relies primarily on fasting and 2-hour post-load plasma glucose concentrations incorporated into current diagnostic criteria [2]. Increasing evidence suggests that this static approach may overlook early abnormalities in glucose regulation and underestimate metabolic risk in a substantial proportion of individuals [3]. Furthermore, longitudinal data indicate that many individuals who subsequently develop type 2 diabetes exhibit normal fasting and 2-hour glucose values at baseline, underscoring the limited sensitivity of conventional OGTT criteria [3–5].

Recent studies have demonstrated that intermediate OGTT time points, particularly 30-minute and 1-hour plasma glucose levels, provide clinically relevant information beyond conventional diagnostic thresholds [6–8]. Early post-load glucose excursions reflect the balance between insulin secretion and insulin sensitivity and are closely linked to β-cell function [7]. Both elevated early glucose levels and delayed time to peak glucose during OGTT have been associated with impaired β-cell responsiveness and an increased risk of progression to prediabetes and type 2 diabetes mellitus (T2DM) [7, 9–11]. In particular, a 1-hour plasma glucose value ≥ 155 mg/dL has been consistently associated with increased risk of future dysglycemia, cardiometabolic complications, and mortality, even among individuals with normal fasting and 2-hour glucose values [3–5, 11, 12]. Similarly, isolated elevations in 30-minute glucose levels have been shown to predict adverse metabolic outcomes independently of conventional OGTT criteria [7, 9, 10, 13]. Despite accumulating evidence, the 1-hour ≥ 155 mg/dL threshold has not yet been incorporated into major clinical diagnostic guidelines and remains under evaluation [6].

Despite the growing recognition of the prognostic value of OGTT-derived glucose dynamics, the biological mechanisms underlying inter-individual variability in glucose responses remain incompletely understood. Prior research has largely focused on genetic determinants and glucose area-under-the-curve metrics; however, a recent systematic review demonstrated that genetic predictors explain only a limited proportion of the observed heterogeneity, with most reported associations being weak, inconsistent, or non-replicable [14]. These findings highlight the need for simple and accessible biochemical markers that may better reflect early metabolic stress captured by OGTT dynamics.

Serum uric acid and albumin are well-established laboratory parameters associated with metabolic and cardiovascular health. Elevated uric acid levels have been linked to oxidative stress, endothelial dysfunction, inflammation, and insulin resistance, whereas serum albumin reflects nutritional status, antioxidant capacity, and systemic inflammatory burden as a negative acute-phase reactant [15–17]. The uric acid-to-albumin ratio (UAR) integrates these opposing pathophysiological processes and has recently been proposed as a composite biomarker reflecting the balance between metabolic burden and protective capacity [16–18]. UAR was selected over single biomarkers because it integrates complementary biological processes (pro-oxidative and anti-oxidative pathways) and is readily available in routine clinical practice, making it a pragmatic candidate for exploratory evaluation.

The rationale for evaluating UAR in relation to OGTT-derived glucose dynamics is based on the potential overlap in underlying pathophysiological processes rather than a direct causal relationship. Early and intermediate glucose excursions during OGTT are primarily driven by impairments in insulin secretion and insulin sensitivity, which are closely linked to oxidative stress and low-grade inflammation [7, 9–11, 19]. These same mechanisms, oxidative stress and low-grade inflammation, have also been associated with alterations in uric acid metabolism and albumin levels [16, 17]. Therefore, UAR may act as an integrated marker reflecting systemic metabolic stress that coincides with, rather than directly determines, early glycemic abnormalities.

Previous studies have demonstrated that UAR is associated with adverse cardiometabolic outcomes, particularly in individuals with established metabolic disease [16–18]. In patients with T2DM, elevated UAR has been independently associated with carotid atherosclerosis and increased mortality risk [16–18]. However, existing studies have primarily focused on overt diabetes and advanced vascular outcomes, and the potential role of UAR in early glycemic dysregulation remains largely unexplored.

To date, no study has systematically examined whether UAR reflects early glycemic disturbances captured by OGTT-derived glucose dynamics prior to the onset of overt diabetes. Addressing this gap may provide novel insights into the pathophysiology of early dysglycemia and help identify individuals at increased metabolic risk who may be misclassified by conventional diagnostic criteria. Therefore, the present study aimed to investigate the association between UAR and dynamic glucose responses during OGTT, with a particular focus on intermediate OGTT time points and mid-OGTT glycemic burden in individuals with normoglycemia and prediabetes.

## Methods

### Study design and population

This retrospective observational study was conducted at a tertiary referral center. A total of 1,500 adult individuals who underwent a standard 75-g OGTT for the evaluation of glucose metabolism between January 2020 and December 2025 were initially screened.

The analyzed dataset consisted of individuals with available OGTT data suitable for glycemic classification. Individuals with conditions that could significantly affect glucose metabolism or uric acid–albumin homeostasis (e.g., chronic kidney disease, active infection, inflammatory disease, malignancy, or use of glucose-lowering medications) were not included in the database prior to analysis. Therefore, no additional exclusions other than diabetes mellitus were applied during the analytical phase.

**Fig. 1:**
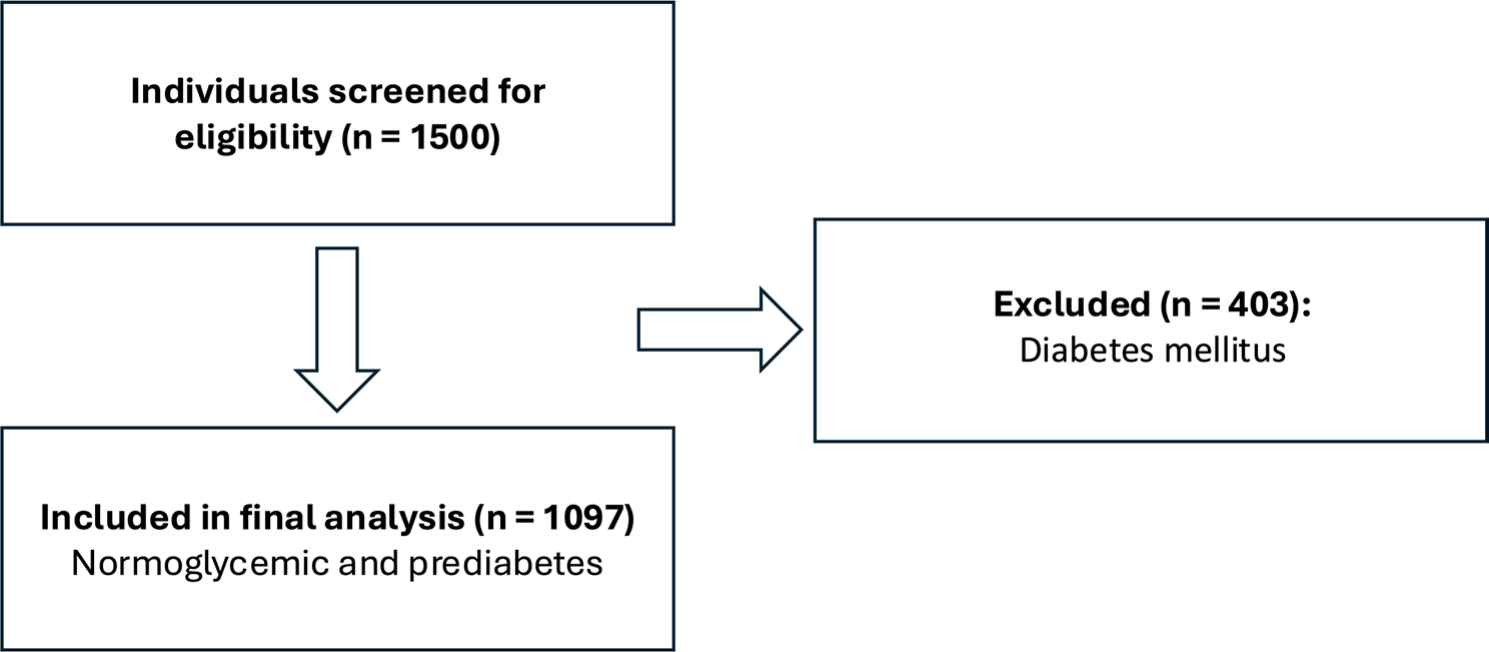
Flow diagram illustrating the study population selection process. A total of 1,500 individuals undergoing a standard 75-g OGTT were screened. The dataset was pre-filtered to exclude individuals with conditions that could significantly affect glucose metabolism or uric acid–albumin homeostasis (e.g., chronic kidney disease, active infection, inflammatory disease, malignancy, or use of glucose-lowering medications). After exclusion of 403 individuals with diabetes mellitus, 1,097 individuals with normoglycemia or prediabetes were included in the final analysis

Of the screened individuals, 403 were identified as having diabetes mellitus and were excluded. The final study population comprised 1,097 individuals with normoglycemia or prediabetes (Fig. 1), classified according to the American Diabetes Association (ADA) criteria [2].

The study protocol was approved by the Gazi University Clinical Research Ethics Committee (Approval No: 2026 − 137, decision date: January 13, 2026) and was conducted in accordance with the principles of the Declaration of Helsinki. Due to the retrospective design of the study, the requirement for written informed consent was waived by the ethics committee.

### OGTT and glycemic classification

All participants underwent a standard 75-g OGTT after an overnight fast of at least 8 hour. Venous plasma glucose concentrations were measured at fasting (0 min) and at 30, 60, 90, and 120 min following glucose ingestion using standardized laboratory methods.

Glycemic status was classified according to ADA diagnostic criteria [2]. Normoglycemia and prediabetes were defined according to the ADA criteria, incorporating fasting plasma glucose, 120-minute plasma glucose, and HbA1c levels. The OGTT-normal subgroup was defined based on fasting plasma glucose < 100 mg/dL and 120-minute plasma glucose < 140 mg/dL. These classifications were based on different diagnostic components.

To evaluate dynamic post-load glucose responses, intermediate OGTT time points (30, 60, and 90 min) were analyzed. Intermediate hyperglycemia was defined as a 60-minute plasma glucose value ≥ 155 mg/dL, based on prior studies demonstrating its association with dysglycemia risk [3–5, 12]. In addition, glucose ≥ 155 mg/dL at any intermediate time point (30, 60, or 90 min) was evaluated as a composite measure of early and mid-phase glycemic excursions.

Participants were further categorized according to the timing of glucose elevation to assess temporal patterns of post-load glucose excursions. Time to peak plasma glucose was defined as the OGTT time point (30, 60, or 90 min) at which the highest plasma glucose concentration was observed. Participants with peak glucose at 0 and 120 min were excluded from time-to-peak analyses, as peak classification was restricted to intermediate OGTT time points (30, 60, and 90 min).

### Laboratory measurements

UAR was calculated by dividing serum uric acid (mg/dL) by serum albumin (g/dL). No additional standardization or transformation was applied.

All biochemical measurements were performed in the central laboratory using automated analyzers integrated with a laboratory information system (Infinity platform), following standardized laboratory procedures. Serum uric acid levels were measured using an enzymatic colorimetric method, and albumin levels were determined using a bromocresol green-based assay.

HbA1c, uric acid, and albumin values were obtained from routine clinical records within a three-month window prior to the OGTT. Laboratory measurements were not uniformly obtained on the same day as the OGTT. When multiple measurements were available within this time window, the value closest to the OGTT date was selected for analysis.

Due to the retrospective design, detailed assay performance characteristics, including intra- and inter-assay coefficients of variation, were not consistently available. However, all measurements were performed under routine internal quality control procedures in the central laboratory.

### Statistical analysis

Statistical analyses were performed using IBM SPSS Statistics, version 31.0 (IBM Corp., Armonk, NY, USA). Continuous variables were assessed for normality using visual methods (histograms and Q–Q plots) and the Kolmogorov–Smirnov test. Normally distributed variables were presented as mean ± standard deviation, whereas non-normally distributed variables were expressed as median (interquartile range). Categorical variables were presented as counts and percentages.

Comparisons between normoglycemic and prediabetic groups were performed using the independent samples t-test for normally distributed variables and the Mann–Whitney U test for non-normally distributed variables. Comparisons across more than two groups (including OGTT-derived subgroups) were conducted using the Kruskal–Wallis test. When the Kruskal–Wallis test was significant, post hoc pairwise comparisons were performed using Bonferroni correction.

Correlations between UAR and OGTT-derived glycemic parameters were evaluated using Spearman’s rank correlation coefficient.

Multivariable logistic regression models were constructed to assess the association of UAR with prediabetes and intermediate OGTT-derived glycemic abnormalities, adjusting for age, sex, BMI, eGFR, triglycerides, and HDL cholesterol. Odds ratios were expressed per 0.1-unit increase in UAR to improve interpretability. Due to missing data in covariates, multivariable analyses were performed in a subset of participants with complete data (complete-case analysis).

OGTT-derived parameters were defined as follows: OGTT AUC was calculated using the trapezoidal method over the 0–120 min period. Late glucose clearance was defined as the difference between intermediate glucose levels and the 120-minute glucose value (60–120 min and 90–120 min). Time to peak glucose was defined as the time point at which the maximum glucose value occurred (30, 60, or 90 min); in cases of ties, the earliest time point was selected.

Receiver operating characteristic (ROC) curve analysis was performed to evaluate the discriminative performance of UAR. Given the multiple comparisons performed, post hoc analyses were adjusted where appropriate, and the results should be interpreted cautiously as exploratory.

All statistical tests were two-sided, and a p-value < 0.05 was considered statistically significant. Given the exploratory nature of baseline comparisons, no formal correction for multiple testing was applied, and these results should be interpreted descriptively.

## Results

### Study population and baseline characteristics

A total of 1,097 individuals were included in the analysis. According to ADA criteria, 176 participants (16.1%) were classified as normoglycemic and 921 (83.9%) as prediabetic.

**Fig. 2:**
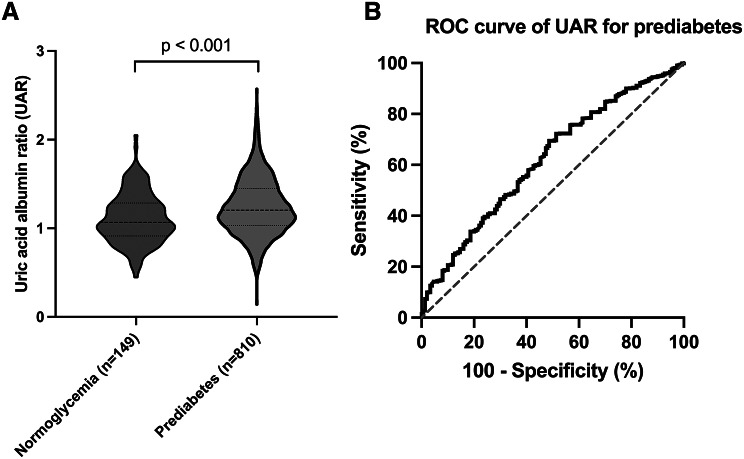
(**A**) Distribution of UAR in normoglycemia and prediabetes groups. Median UAR values were higher in the prediabetes group compared with the normoglycemia group (*p* < 0.001). (**B**) Receiver operating characteristic (ROC) curve of UAR for prediabetes. The area under the curve (AUC) was 0.62 (95% Cl 0.57–0.67, *p* < 0.001), indicating modest discriminative ability. This analysis is presented as exploratory

Baseline demographic and biochemical characteristics according to glycemic status are summarized in Table 1. Individuals with prediabetes were significantly older than normoglycemic individuals. Median HbA1c levels were higher in the prediabetic group. Serum uric acid concentrations and UAR were also significantly elevated among individuals with prediabetes (Fig. 2A), whereas serum albumin levels did not differ significantly between groups.

**Table 1:** Baseline characteristics of the study population according to glycemic status (ADA criteria)

Variable	Normoglycemia	Prediabetes	*p*
Age, years (*n* = 176 / 921)	35 (28–46)	50 (41–59)	< 0.001
Female sex, n (%)	131 (74.4)	646 (70.1)	0.251
Male sex, n (%)	45 (25.6)	275 (29.9)	
Uric acid, mg/dL (*n* = 149 / 823)	4.80 (4.10–5.80)	5.30 (4.50–6.40)	< 0.001
Albumin, g/dL (*n* = 154 / 817)	4.50 (4.30–4.70)	4.45 (4.25–4.55)	0.070
UAR (uric acid/albumin) (*n* = 149 / 810)	1.07 (0.91–1.29)	1.20 (1.03–1.45)	< 0.001
HbA1c, % (*n* = 176 / 901)	5.40 (5.20–5.50)	6.00 (5.80–6.20)	< 0.001
Creatinine, mg/dL (*n* = 88 / 313)	0.67 (0.58–0.82)	0.71 (0.63–0.85)	0.024
eGFR, mL/min/1.73 m² (*n* = 88 / 313)	115.6 (106.6–121.7)	104.8 (97.7–111.4)	< 0.001
BMI, kg/m² (*n* = 48 / 407)	26.5 (23.5–32.4)	30.2 (26.0–35.3)	0.002
Triglycerides, mg/dL (*n* = 64 / 514)	107 (68–156)	128 (95–176)	0.001
Total cholesterol, mg/dL (*n* = 64 / 513)	190 (164–213)	206 (178–234)	0.003
LDL cholesterol, mg/dL (*n* = 64 / 512)	114 (98–137)	127 (104–149)	0.018
HDL cholesterol, mg/dL (*n* = 64 / 512)	51 (39–59)	48 (40–58)	0.639

Data are presented as median (interquartile range) or number (percentage), as appropriate

n values indicate the number of participants with available data for each variable

Due to missing data, sample sizes vary across variables

Glycemic status was defined according to ADA criteria using fasting glucose, 120-minute OGTT glucose, and HbA1c

### OGTT-derived glucose dynamics and intermediate hyperglycemia

OGTT-derived plasma glucose values are presented in Table 2. Across all OGTT time points, glucose concentrations were significantly higher in individuals with prediabetes (all *p* < 0.001).

**Table 2:** OGTT-derived plasma glucose levels by glycemic status

Parameter	Normoglycemic (*n* = 176)	Prediabetic (*n* = 921)	*P* value
Fasting glucose (mg/dL)	87.7 (82.0–93.2)	98.2 (90.0–105.4)	< 0.001
30-min glucose (mg/dL)	130.9 (118.2–147.8)	148.7 (133.5–167.2)	< 0.001
60-min glucose (mg/dL)	130.8 (105.4–152.7)	163.3 (133.6–192.7)	< 0.001
90-min glucose (mg/dL)	107.7 (90.2–126.8)	142.0 (112.8–171.0)	< 0.001
120-min glucose (mg/dL)	99.1 (83.9–114.6)	120.9 (100.7–146.6)	< 0.001

Data are presented as median (interquartile range)

The prevalence of intermediate hyperglycemia, defined as a 60-minute plasma glucose value ≥ 155 mg/dL, is shown in Table 3 and was significantly higher in the prediabetic group compared with normoglycemic individuals (58.0% vs. 22.9%, *p* < 0.001). This difference remained significant within the OGTT-normal subgroup (31.4% vs. 22.9%, *p* = 0.036) (Table 3).

**Table 3:** Prevalence of dynamic glycemic abnormalities based on OGTT thresholds

Parameter	Normoglycemic (*n* = 176)	Prediabetic (*n* = 921)	*P* value
60-min glucose ≥ 155 mg/dL (intermediate hyperglycemia, overall cohort), n (%)	40 (22.9%)	534 (58.0%)	< 0.001
60-min glucose ≥ 155 mg/dL (intermediate hyperglycemia, OGTT-normal subgroup), n (%)	40 (22.9%)	127 (31.4%)	0.036
Glucose ≥ 155 mg/dL at any intermediate time point (30, 60, or 90 min; overall cohort), n (%)	52 (29.5%)	615 (66.8%)	< 0.001
Glucose ≥ 155 mg/dL at any intermediate time point (30, 60, or 90 min; OGTT-normal subgroup), n (%)	52 (29.5%)	168 (41.5%)	0.006

Data are presented as number (percentage)

Intermediate hyperglycemia was defined as a 60-minute plasma glucose value ≥ 155 mg/dL

Glucose ≥ 155 mg/dL at any intermediate time point (30, 60, or 90 min) was also evaluated. The OGTT-normal subgroup was defined as fasting plasma glucose < 100 mg/dL and 120-minute plasma glucose < 140 mg/dL

In addition, glucose ≥ 155 mg/dL at any intermediate time point (30, 60, or 90 min) was also more frequently observed in individuals with prediabetes than in normoglycemic individuals (66.8% vs. 29.5%, *p* < 0.001), including within the OGTT-normal subgroup (41.5% vs. 29.5%, *p* = 0.006) (Table 3).

### Association of UAR with OGTT glucose dynamics

UAR showed significant positive correlations with multiple OGTT-derived glycemic parameters. These included early glucose excursion, delayed time to peak glucose, higher OGTT area under the curve, and the presence of intermediate glucose elevations (Table 4). Correlations with late-phase glucose clearance were modest but statistically significant. These associations remained directionally consistent when analyses were stratified by glycemic status (Supplementary Table 6).

**Table 4:** Association of UAR with OGTT-derived glycemic dynamics and glucose excursions

OGTT parameter	Spearman ρ	*p*
Δ glucose (30–0 min)	0.099	0.002
Time to maximum glucose	0.190	< 0.001
Any intermediate time glucose ≥ 155 mg/dL	0.212	< 0.001
Late glucose clearance (60–120 min)	0.094	0.004
Late glucose clearance (90–120 min)	0.152	< 0.001
OGTT AUC	0.274	< 0.001

Data are presented as Spearman correlation coefficients (ρ). Δ glucose (30–0 min) was calculated as the difference between 30-minute and fasting plasma glucose. Time to maximum glucose was defined as the OGTT time point (30, 60, or 90 min) at which peak glucose occurred. Late glucose clearance was defined as the difference between glucose levels at 60–90 min and 120 min. OGTT AUC represents the total area under the glucose curve during the 0–120 min OGTT. Glucose ≥ 155 mg/dL at any intermediate time point refers to plasma glucose values ≥ 155 mg/dL at 30, 60, or 90 min

Among OGTT-normal individuals, those exhibiting plasma glucose values ≥ 155 mg/dL at any intermediate OGTT time point had significantly higher UAR values compared with those without such excursions (*p* = 0.003) (Table 5). Similarly, in the overall study population, individuals with intermediate glucose ≥ 155 mg/dL demonstrated higher UAR values than those without such elevations (*p* < 0.001) (Table 5).

**Table 5:** UAR levels according to 60-minute glucose-defined intermediate hyperglycemia and intermediate OGTT glucose elevations

Population	Glycemic category	*n* (%)	UAR (median, IQR)	*p* value
OGTT-normal subgroup	60-min glucose < 155 mg/dL	412 (71.2%)	1.1076 (0.9432–1.3012)	< 0.001
	60-min glucose ≥ 155 mg/dL (intermediate hyperglycemia)	167 (28.8%)	1.1868 (1.0549–1.4607)	
Overall cohort	60-min glucose < 155 mg/dL	521 (47.6%)	1.1222 (0.9444–1.3170)	< 0.001
	60-min glucose ≥ 155 mg/dL (intermediate hyperglycemia)	574 (52.4%)	1.2584 (1.0602–1.5062)	
OGTT-normal subgroup	No elevation at any intermediate time point	361 (62.1%)	1.1111 (0.9438–1.3237)	0.003
	Glucose ≥ 155 mg/dL at any intermediate time point (30, 60, or 90 min)	220 (37.9%)	1.1585 (1.0390–1.4194)	
Overall cohort	No elevation at any intermediate time point	430 (39.2%)	1.1160 (0.9425–1.3103)	< 0.001
	Glucose ≥ 155 mg/dL at any intermediate time point (30, 60, or 90 min)	667 (60.8%)	1.2414 (1.0538–1.4839)	

Data are presented as median (interquartile range)

Intermediate hyperglycemia was defined as a 60-minute plasma glucose value ≥ 155 mg/dL

Glucose ≥ 155 mg/dL at any intermediate time point (30, 60, or 90 min) was analyzed separately as an additional OGTT-derived measure

The OGTT-normal subgroup was defined as fasting plasma glucose < 100 mg/dL and 120-minute plasma glucose < 140 mg/dL

In addition, UAR levels differed according to the timing of peak plasma glucose during OGTT. Individuals reaching peak glucose at later time points exhibited a monotonic increase in UAR values, with significant differences observed between the 30-, 60-, and 90-minute peak groups (Supplementary Table 7).

### UAR and glycemic outcomes

Multivariable logistic regression analyses were performed in a complete-case subset of participants with available covariate data (*n* = 378), reflecting a reduced sample size compared to the overall cohort due to missingness in key covariates. UAR was not significantly associated with prediabetes (OR: 1.10 per 0.1-unit increase, 95% CI: 0.94–1.28, *p* = 0.228) (Supplementary Table 1).

In contrast, when intermediate hyperglycemia was defined using the established 60-minute plasma glucose threshold (≥ 155 mg/dL), UAR was independently associated with increased odds of intermediate hyperglycemia. Each 0.1-unit increase in UAR was associated with a 1.13-fold higher odds of 60-minute glucose ≥ 155 mg/dL (OR: 1.13, 95% CI: 1.03–1.24, *p* = 0.006), independent of age, sex, BMI, eGFR, triglycerides, and HDL cholesterol (Supplementary Table 3).

A similar association was observed when intermediate hyperglycemia was defined as glucose ≥ 155 mg/dL at any intermediate time point (30, 60, or 90 min), although the magnitude of the association was smaller (OR: 1.10 per 0.1-unit increase, 95% CI: 1.01–1.20, *p* = 0.029) (Supplementary Table 2).

When UAR was categorized into quartiles, only the highest quartile was significantly associated with increased odds of 60-minute glucose ≥ 155 mg/dL (Supplementary Table 4). A similar pattern was observed for glucose ≥ 155 mg/dL at any intermediate time point (30, 60, or 90 min), although the overall association across quartiles was not statistically significant (Supplementary Table 5). A summary of data availability across key covariates is presented in Supplementary Table 8.

### Receiver operating characteristic (ROC) analysis

ROC analysis demonstrated that UAR demonstrated limited discriminative ability in relation to prediabetes (AUC = 0.62) (Fig. 2B). Using the Youden index, a UAR threshold of 1.051 was identified (sensitivity ~ 72%, specificity ~ 52%); however, this threshold should be interpreted as descriptive and exploratory rather than clinically applicable, pending external validation.

## Discussion

In this study, UAR was associated with early and intermediate disturbances in glucose metabolism during OGTT, whereas its association with conventionally defined prediabetes was not independent after adjustment. Importantly, these associations extended beyond conventional fasting and 2-hour glucose-based classifications and were evident in individuals exhibiting intermediate hyperglycemia during the early and mid phases of the OGTT. These findings indicate that UAR may reflect early post-load glycemic dysregulation not reflected by conventional fasting and 2-hour glucose measures.

The relationship between UAR and OGTT-derived glycemic abnormalities may represent early metabolic stress preceding overt dysglycemia. Uric acid has been linked to oxidative stress, low-grade inflammation and endothelial dysfunction, whereas albumin contributes substantially to circulating antioxidant capacity; thus, their ratio may capture a composite signal of metabolic stress linking oxidative imbalance and inflammatory activation [15–18]. Accumulating evidence indicates that early disturbances in glucose metabolism are primarily driven by impairments in first-phase insulin secretion and delayed post-load glucose clearance processes that are not adequately reflected by fasting or 2-hour plasma glucose measurements alone [4, 9]. Accordingly, intermediate OGTT time points, including 30-, 60- and 90-minute glucose levels, peak glucose concentration and overall glucose excursion patterns, have been shown to better represent early β-cell dysfunction and insulin resistance [7, 10, 11].

In this context, elevated UAR levels may capture metabolic alterations that coincide with early post-load glycemic abnormalities, even among individuals who do not meet conventional criteria for prediabetes. This interpretation is biologically plausible given prior reports demonstrating that early post-challenge hyperglycemia is associated with inflammatory activation, oxidative stress and impaired insulin dynamics-mechanisms that may also influence uric acid metabolism and albumin homeostasis [5, 19, 20]. Collectively, these observations suggest that UAR may represent an integrated marker of early metabolic stress that aligns more closely with OGTT-derived intermediate glycemic disturbances than with static glucose measurements alone. In line with this interpretation, delayed time to peak glucose during OGTT has been proposed as a dynamic marker of early β-cell dysfunction and impaired insulin sensitivity [7]. Chung et al. demonstrated that a glucose peak occurring beyond 30 min was independently associated with prediabetes risk and reduced disposition index, supporting the relevance of peak timing as an indicator of early metabolic stress [7]. This finding is particularly relevant, as it suggests that individuals classified as normoglycemic by conventional criteria may already exhibit measurable metabolic stress potentially reflected by UAR.

Although the discriminative performance of UAR was limited, its association with intermediate glycemic abnormalities not captured by conventional fasting and 2-hour glucose criteria highlights its potential role as a complementary metabolic marker. Similar modest discriminative performance has been reported for other early metabolic risk indicators derived from OGTT dynamics, such as 1-hour plasma glucose and peak glucose timing, which are primarily used for risk stratification rather than diagnosis [11, 12, 21]. In this context, UAR may provide incremental information when interpreted alongside OGTT-derived measures, particularly in relation to early or intermediate glycemic disturbances [9]. In line with this, effect estimates expressed per 0.1-unit increase in UAR demonstrated modest but statistically significant associations with intermediate hyperglycemia, indicating that relatively small differences in UAR may have clinical relevance. Notably, after adjustment for metabolic and renal confounders, UAR was not independently associated with prediabetes. However, UAR remained independently associated with intermediate hyperglycemia when defined using the established 60-minute plasma glucose threshold (≥ 155 mg/dL), which has the strongest evidence base in the literature. In secondary analyses, UAR was also associated with a broader composite definition of intermediate hyperglycemia (glucose ≥ 155 mg/dL at any intermediate OGTT time point: 30, 60, or 90 min), although this composite may reflect heterogeneous glycemic patterns. These findings suggest that UAR may reflect early dynamic disturbances in glucose metabolism rather than conventional glycemic classifications based on fasting and 2-hour glucose values. However, the magnitude of these associations was moderate, indicating limited standalone clinical applicability. Notably, the association between UAR and intermediate hyperglycemia did not appear to be strictly linear. When UAR was analyzed in quartiles, only the highest category was associated with increased risk, suggesting a potential threshold effect.

Previous studies have linked uric acid-related markers, including UAR, to insulin resistance, prediabetes and adverse cardiometabolic outcomes, largely focusing on static glycemic measures or advanced disease stages [16–18]. In parallel, a growing body of evidence underscores the clinical relevance of OGTT-derived glycemic dynamics in capturing early β-cell dysfunction and delayed glucose clearance that remain undetected by fasting or 2-hour glucose values alone [3, 11, 12]. Building on this literature, the present study integrates a routinely available biochemical marker with detailed OGTT time-point analyses in a large cohort. To date, evidence on the relationship between UAR and OGTT-derived glycemic dynamics remains limited. In this study, we show that UAR is associated with intermediate OGTT hyperglycemia and peak-timing patterns beyond conventional criteria, thereby extending the potential role of UAR from a marker associated with advanced metabolic or cardiovascular outcomes to one linked to early and intermediate disturbances in glucose metabolism.

The present study has several strengths, including a relatively large cohort, the availability of multiple OGTT time points enabling detailed assessment of dynamic glycemic responses, and the evaluation of a simple, routinely available biochemical marker. Nevertheless, the retrospective, single-center design precludes causal inference and may limit generalizability. The high prevalence of prediabetes likely reflects referral bias inherent to a tertiary care setting. In addition, the applicability of the ≥ 155 mg/dL threshold across different populations remains uncertain. The smaller size of the 90-minute subgroup may have limited statistical power. Multivariable analyses were performed in a reduced sample due to missing data in key covariates, and laboratory parameters obtained within a three-month window may have introduced temporal variability. Furthermore, important determinants of uric acid metabolism, including dietary factors, medication use, blood pressure, smoking, alcohol consumption, and socioeconomic variables, were not consistently available, potentially resulting in residual confounding. The observed positive association between eGFR and prediabetes is counterintuitive and may reflect selection bias and the relatively narrow distribution of renal function in this cohort rather than a true biological relationship. Overall, this study should be considered hypothesis-generating, and the findings require confirmation in prospective studies.

From a clinical perspective, UAR may provide complementary metabolic information in relation to early OGTT-derived glycemic disturbances; however, its standalone discriminative performance is modest and should be interpreted with caution. Given the observed pattern of association, UAR may reflect underlying metabolic processes related to early glucose dysregulation, but should be considered a complementary rather than a standalone marker. Further prospective studies are required to determine its potential role in future dysglycemia.

## Electronic Supplementary Material

Below is the link to the electronic supplementary material.

Supplementary Material 1

## Data Availability

The datasets generated and/or analyzed during the current study are not publicly available due to institutional and ethical restrictions related to patient confidentiality. However, de-identified data can be made available from the corresponding author upon reasonable request and with appropriate institutional approval.

## Abbreviations

OGTT: Oral glucose tolerance test
UAR: Uric acid to albumin ratio
ADA: American Diabetes Association
T2DM: Type 2 diabetes mellitus
AUC: Area under the curve
ROC: Receiver operating characteristic
BMI: Body mass index
eGFR: Estimated glomerular filtration rate
HDL: High-density lipoprotein
LDL: Low-density lipoprotein
HbA1c: Glycated hemoglobin
