# Supplementary material for: Association of the uric acid to albumin ratio with early glycemic disturbances beyond conventional OGTT criteria
Source: BMC Endocr Disord. 2026 May 12;26:196. doi: 10.1186/s12902-026-02314-x (PMC13334900; doi:10.1186/s12902-026-02314-x)
Supplement: Supplementary file 1 — Supplementary Material 1 [file 12902_2026_2314_MOESM1_ESM.docx]

**Supplementary Material**

**Multivariable logistic regression analyses for prediabetes and intermediate glucose excursions**

| **Supplementary Table 1. Model 1 Prediabetes** | | |
| --- | --- | --- |
| **Variable** | **OR (95% CI)** | **p-value** |
| Age | 1.24 (1.11–1.38) | <0.001 |
| UAR (per 0.1-unit increase) | 1.10 (0.94–1.28) | 0.228 |
| Sex (male) | 0.77 (0.30–2.00) | 0.592 |
| BMI | 1.06 (1.00–1.12) | 0.056 |
| eGFR | 1.25 (1.08–1.45) | 0.003 |
| Triglycerides | 1.00 (0.99–1.01) | 0.642 |
| HDL-C | 0.99 (0.96–1.03) | 0.711 |

**Model statistics:**

- n = 378
- χ² = 33.1, p < 0.001
- Nagelkerke R² = 0.177

| **Supplementary Table 2. Model 2 Glucose ≥155 mg/dL at any intermediate time point (30, 60, or 90 min)** | | |
| --- | --- | --- |
| **Variable** | **OR (95% CI)** | **p-value** |
| Age | 1.16 (1.04–1.30) | 0.007 |
| UAR (per 0.1-unit increase) | 1.10 (1.01–1.20) | 0.029 |
| Sex (male) | 1.65 (0.88–3.08) | 0.121 |
| BMI | 1.04 (1.00–1.08) | 0.040 |
| eGFR | 1.20 (1.02–1.41) | 0.029 |
| Triglycerides | 1.01 (1.00–1.01) | 0.001 |
| HDL-C | 1.01 (0.99–1.03) | 0.586 |

**Model statistics:**

- n = 378
- χ² = 55.3, p < 0.001
- Nagelkerke R² = 0.186

| **Supplementary Table 3. Model 3 Multivariable logistic regression analysis for 60-minute plasma glucose ≥155 mg/dL (intermediate hyperglycemia)** | | |
| --- | --- | --- |
| **Variable** | **OR (95% CI)** | **p-value** |
| Age | 1.17 (1.04–1.30) | 0.013 |
| UAR (per 0.1-unit increase) | 1.13 (1.03–1.24) | 0.006 |
| Sex (male) | 1.60 (0.85–3.02) | 0.136 |
| BMI | 1.05 (1.01–1.09) | 0.013 |
| eGFR | 1.22 (1.02–1.46) | 0.034 |
| Triglycerides | 1.01 (1.00–1.01) | 0.002 |
| HDL-C | 1.00 (0.98–1.02) | 0.943 |
| Intermediate hyperglycemia was defined as 60-minute plasma glucose ≥155 mg/dL during OGTT. All models were adjusted for age, sex, BMI, eGFR, triglycerides, and HDL cholesterol. UAR: uric acid-to-albumin ratio. | | |

**Model statistics:**

- n = 377
- χ² = 58.9, p < 0.001
- Nagelkerke R² = 0.193

| **Supplementary Table 4. Multivariable logistic regression analysis of UAR quartiles for predicting 60-minute plasma glucose ≥155 mg/dL** | | | |
| --- | --- | --- | --- |
| **UAR Quartile** | **OR** | **95% CI** | **p-value** |
| Q1 (lowest) | 1.00 | Reference | – |
| Q2 | 1.23 | (0.65–2.32) | 0.523 |
| Q3 | 1.46 | (0.76–2.82) | 0.256 |
| Q4 (highest) | 2.52 | (1.24–5.11) | 0.010 |
| Adjusted for age, sex, BMI, eGFR, triglycerides, and HDL cholesterol. | | | |

| **Supplementary Table 5: Multivariable logistic regression analysis of UAR quartiles for predicting glucose ≥155 mg/dL at any intermediate time point (30, 60, or 90 minutes)** | | | |
| --- | --- | --- | --- |
| **UAR Quartile** | **OR** | **95% CI** | **p-value** |
| Q1 (lowest) | 1.00 | Reference | – |
| Q2 | 1.22 | (0.65–2.29) | 0.543 |
| Q3 | 1.31 | (0.74–2.33) | 0.429 |
| Q4 (highest) | 2.14 | (1.03–4.45) | 0.041 |
| Adjusted for age, sex, BMI, eGFR, triglycerides, and HDL cholesterol. | | | |

| **Supplementary Table 6. Correlation of UAR with OGTT-derived parameters stratified by glycemic status** | | | | |
| --- | --- | --- | --- | --- |
| **OGTT parameter** | **Normoglycemia (r)** | **p-value** | **Prediabetes (r)** | **p-value** |
| Δ glucose (30–0 min) | 0.008 | 0.918 | -0.068 | 0.039 |
| Time to maximum glucose | 0.210 | 0.006 | 0.371 | <0.001 |
| Any intermediate glucose ≥155 mg/dL | 0.636 | <0.001 | 0.532 | <0.001 |
| Late glucose clearance (60–120 min) | 0.588 | <0.001 | 0.509 | <0.001 |
| Late glucose clearance (90–120 min) | 0.450 | <0.001 | 0.405 | <0.001 |
| OGTT AUC | 0.713 | <0.001 | 0.556 | <0.001 |
| Correlations were assessed using Spearman’s rank correlation coefficient. Δ glucose (30–0 min): difference between 30-minute and fasting glucose. Time to maximum glucose: time point (30, 60, or 90 minutes) at which peak glucose occurs. Late glucose clearance (60–120 min): difference between 60-minute and 120-minute glucose. Late glucose clearance (90–120 min): difference between 90-minute and 120-minute glucose. OGTT AUC: total glucose area under the curve during 0–120 minutes. Any intermediate glucose ≥155 mg/dL: plasma glucose ≥155 mg/dL at any intermediate OGTT time point (30, 60, or 90 minutes). | | | | |

| **Supplementary Table 7. Time to peak plasma glucose during OGTT and UAR levels with pairwise comparisons** | | | |
| --- | --- | --- | --- |
| **Time to peak glucose during OGTT** | **n (%)** | **UAR** | **Pairwise comparison (p)** |
| **30 min** | 377 (35.2%) | 1.11 (0.94–1.30) | vs 60 min: **<0.001** vs 90 min: **<0.001** |
| **60 min** | 597 (55.7%) | 1.23 (1.03–1.47) | vs 90 min: **0.042** |
| **90 min** | 97 (9.1%) | 1.27 (1.13–1.54) | — |
| Data are presented as median (interquartile range) or percentage. Comparisons across groups were performed using the Kruskal–Wallis test. Post hoc pairwise comparisons were conducted with Bonferroni correction. | | | |

| **Supplementary Table 8. Summary of data availability across key covariates** | | | |
| --- | --- | --- | --- |
| **Variable** | **Available (n)** | **Missing (n)** | **Missing (%)** |
| BMI | 455 | 642 | 58.5% |
| eGFR | 401 | 696 | 63.4% |
| Triglycerides | 578 | 519 | 47.3% |
| HDL cholesterol | 576 | 521 | 47.5% |
| Total sample size: n = 1,097. Missing values were calculated based on the number of participants with available data for each variable. | | | |
